# Bioinformatic Mining and Structure-Activity Profiling of Baeyer-Villiger Monooxygenases from Mycobacterium tuberculosis

**DOI:** 10.1128/msphere.00482-21

**Published:** 2022-03-17

**Authors:** Nicolas Tomas, Dimitri Leonelli, Martin Campoy, Sylvain Marthey, Nguyen-Hung Le, David Rengel, Véronique Martin, Adrian Pál, Jana Korduláková, Nathalie Eynard, Valérie Guillet, Lionel Mourey, Mamadou Daffé, Anne Lemassu, Gwenaëlle André, Hedia Marrakchi

**Affiliations:** a Institut de Pharmacologie et de Biologie Structurale, IPBS, Université de Toulouse, CNRS, UPS, Toulouse, France; b MaIAGE, INRAE, AgroParisTech, Université Paris-Saclay, Jouy-en-Josas, France; c Department of Biochemistry, Faculty of Natural Sciences, Comenius University in Bratislava, Bratislava, Slovakia; d LMGM-CBI, UMR 5100, Université de Toulouse, CNRS, UPS, Toulouse, France; Washington University School of Medicine in St. Louis

**Keywords:** tuberculosis, mycobacteria, monooxygenase, BVMO signature, modeling, substrate selectivity, protein structure-function

## Abstract

Mycobacterium tuberculosis is the etiological agent of tuberculosis (TB), one of the deadliest infectious diseases. The alarming health context coupled with the emergence of resistant M. tuberculosis strains highlights the urgent need to expand the range of anti-TB antibiotics. A subset of anti-TB drugs in use are prodrugs that require bioactivation by a class of M. tuberculosis enzymes called Baeyer-Villiger monooxygenases (BVMOs), which remain understudied. To examine the prevalence and the molecular function of BVMOs in mycobacteria, we applied a comprehensive bioinformatic analysis that identified six BVMOs in M. tuberculosis, including Rv3083 (MymA), Rv3854c (EthA), Rv0565c, and Rv0892, which were selected for further characterization. Homology modeling and substrate docking analysis, performed on this subset, suggested that Rv0892 is closer to the cyclohexanone BVMO, while Rv0565c and EthA are structurally and functionally similar to MymA, which is by far the most prominent type I BVMO enzyme. Thanks to an unprecedented purification and assay optimization, biochemical studies confirmed that all four BVMOs display BV-oxygenation activity. We also showed that MymA displays a distinctive substrate preference that we further investigated by kinetic parameter determination and that correlates with *in silico* modeling. We provide insights into distribution of BVMOs and the structural basis of their substrate profiling, and we discuss their possible redundancy in M. tuberculosis, raising questions about their versatility in prodrug activation and their role in physiology and infection.

**IMPORTANCE** Tuberculosis (TB), caused by Mycobacterium tuberculosis, is one of the leading causes of death worldwide. The rise in drug resistance highlights the urgent need for innovation in anti-TB drug development. Many anti-TB drugs require bioactivation by Baeyer-Villiger monooxygenases (BVMOs). Despite their emerging importance, BVMO structural and functional features remain enigmatic. We applied a comprehensive bioinformatic analysis and confirmed the presence of six BVMOs in M. tuberculosis, including MymA, EthA, and Rv0565c—activators of the second-line prodrug ethionamide—and the novel BVMO Rv0892. Combining *in silico* characterization with *in vitro* validation, we outlined their structural framework and substrate preference. Markedly, MymA displayed an enhanced capacity and a distinct selectivity profile toward ligands, in agreement with its catalytic site topology. These features ground the molecular basis for structure-function comprehension of the specificity in these enzymes and expand the repertoire of BVMOs with selective and/or overlapping activity for application in the context of improving anti-TB therapy.

## INTRODUCTION

Tuberculosis (TB) caused by Mycobacterium tuberculosis remains a major health problem worldwide with 10 million people infected and more than 1.5 million deaths per year (1). Despite the existence of antibiotics and a vaccine, the treatment of TB remains complex, especially in the context of comorbidity factors such as coinfection with human immunodeficiency virus (HIV)/AIDS, the recent SARS-CoV-2 virus, diabetes, and broader determinants such as poverty, undernutrition, and poor access to cures in low-income countries. The emergence of multidrug-resistant TB (MDR-TB) and extremely resistant TB (XDR-TB) adds to this global health issue. Therefore, there is an urgent need to develop new anti-TB drugs that would shorten the therapy duration, improve efficacy against MDR-TB, and enable administration of drug combinations with antiretroviral agents.

One strategy to accelerate development of anti-TB compounds is based on exploiting molecules and mechanisms whose effectiveness is already established. Among the drugs known to be effective, several must be bioactivated by M. tuberculosis to become highly toxic to the pathogen. This is particularly the case for ethionamide (ETH), isoxyl (ISO), and thiacetazone (TAC), known anti-TB prodrugs, but also newly identified compounds active against M. tuberculosis; all are activated through oxidation by a peculiar class of monooxygenases, the Baeyer-Villiger monooxygenases (BVMOs) (2, 3). Indeed, the M. tuberculosis genome includes at least three BVMO genes that can activate ETH: *rv3854c* (also known as *ethA* for ETH activator) (4), *rv3083* (also known as *mymA*) (5), and *rv0565c* (6). EthA and MymA are both type I BVMOs, which means that they both harbor a Rossmann fold and are dependent on flavin adenine dinucleotide (FAD) cofactor and NADPH electron donor (7). Moreover, both enzymes have been recognized as important for developing anti-M. tuberculosis inhibitors with improved efficacy and reduced toxicity (3, 5). Phenotypic analyses of *ethA* and *mymA* deletion mutants strongly indicate their involvement in lipid metabolism, including metabolism of mycolic acids (8–10). Also, the *mymA* operon is required for proper cell envelope ultrastructure and ability to survive inside activated macrophages (9). Yet, the prevalence and distribution of BVMOs in mycobacteria are still unknown, and their physiological function has not been explored in depth. Thus, it is of interest to identify and profile structurally and functionally the BVMOs of this phylum.

Here, we conducted a systematic bioinformatic analysis of 12 actinobacterial genomes, including those from mycobacterial species (M. tuberculosis, M. leprae, M. smegmatis, M. bovis, M. marinum, enlarged to M. phlei, and M. vaccae) and the *Mycobacteriales* order (Corynebacterium glutamicum, Nocardia farcinica, Rhodococcus jostii, Mycolicibacterium gilvum, and Hoyosella subflava) to characterize prevalence and distribution of type I BVMOs. Among the six putative BVMOs distributed along the phylogenic tree in M. tuberculosis, we selected the candidate BVMOs Rv0565c and Rv0892, which exhibited sequence and structure similarity to MymA and EthA, as supported by homology modeling and substrate docking. The *in silico* results were further confirmed by *in vitro* assays, which showed that all four BVMOs can accommodate a variety of molecules. If Rv0892 is the most restricted enzyme in regard to substrate selectivity, Rv0565c, EthA, and MymA evidence a rather similar substrate profiling, with a particularly effective activity for MymA onto ethyl 3-oxohexanoate (Et3-ox). Such substrate acceptance is discussed in terms of sequence/structure features and promiscuity required for enzyme evolution as well as overlapping activity for application in the context of improving anti-TB therapy.

## RESULTS

### Sequence analysis and three-dimensional (3D) structure of M. tuberculosis BVMOs.

**FIG 1:**
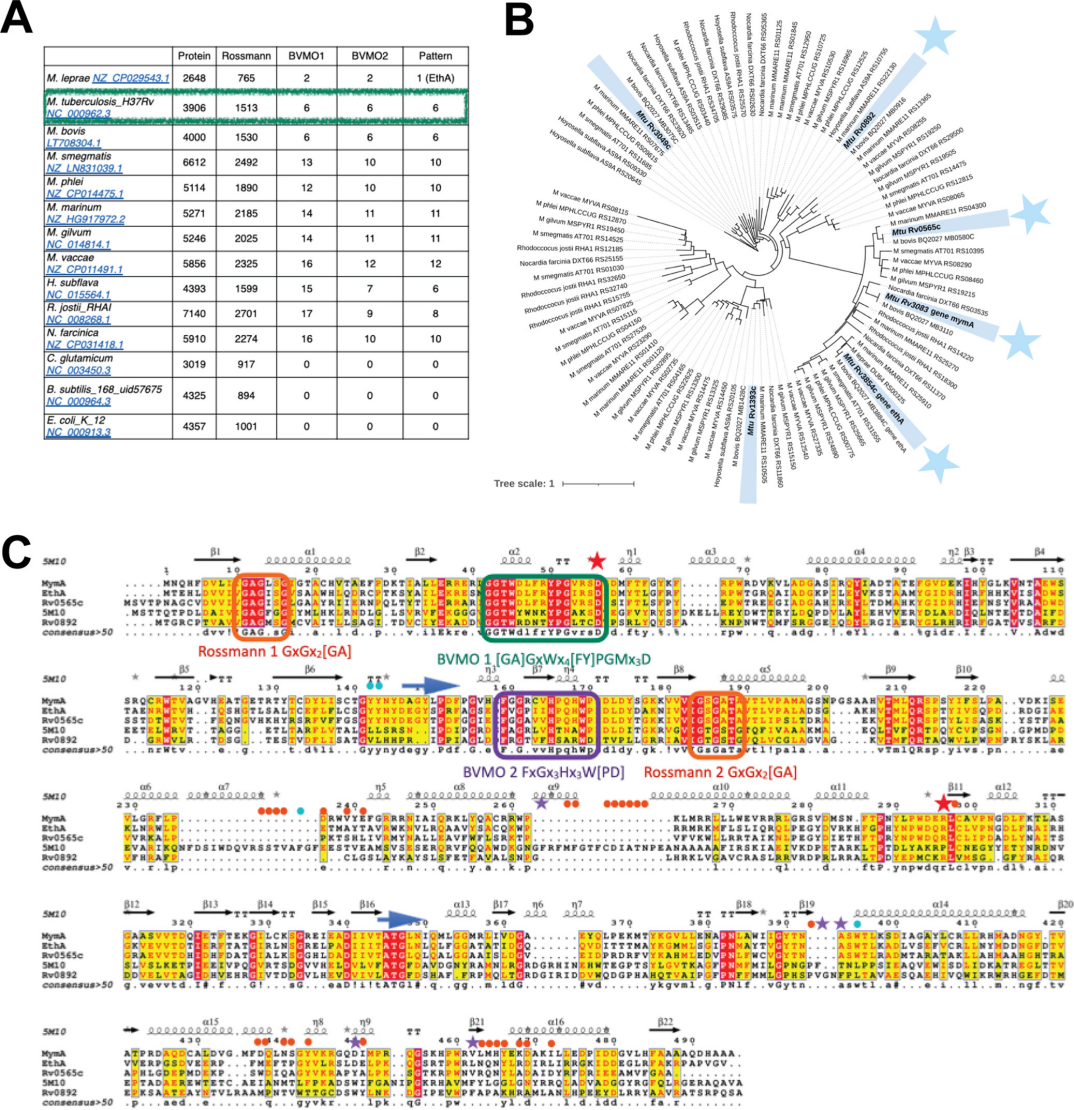
Distribution and structural features of BVMOs. (A) Cooccurrence of motifs that form specific fingerprints and characterize type I BVMOs as observed within 12 actinobacterial and selected Gram-positive and Gram-negative reference genomes. Fingerprints have been identified using FuzzPro (EBI) and in-house interactive visualization implemented under the COSP workflow (https://github.com/SmartBioInf/COSP). (B) Phylogenetic tree from the putative type I BVMO using NGphylogeny and iTOL. (C) Multiple alignment of the four selected BVMOs with the 3D template *Tm*CHMO, using ClustalW and ESPript. The pattern motifs contoured in orange, deep teal, and purple are Rossmann 1 and 2, BVMO1, and BVMO2, respectively. The catalytic aspartate and arginine residues are marked with red stars, and the residues that define the active site are highlighted with a cyan circle, while the ones that define the substrate tunnel have a solid orange circle, and those that could restrict the entrance between the substrate tunnel and the active site are highlighted with a purple star. The two blue arrows define the hinge linkers that connect the FAD and NADPH domains and are involved in domain rotation.

In order to gain insight into prevalence and distribution of BVMOs in M. tuberculosis, we conducted a bioinformatic analysis for 12 actinobacteria and two reference genomes (Escherichia coli and Bacillus subtilis), with accession genomes listed in Fig. 1A. The numbering of BVMOs is reported in Fig. 1A, and details of the identification are available on the GitHub page (https://smartbioinf.github.io/BVMO_type1_Tomas_et_al_2021/). The identified sequences strictly display the hallmark of BVMO, which is composed of BVMO1 and BVMO2 fingerprints [GA]GxWx_4_[FY]P[GM]x_3_D and FxGx_3_Hx_3_W[PD], respectively, encompassed by 2 Rossmann motifs composed of GxGx_2_[GA]. Markedly, we implemented a visualization pipeline which evidences that the BVMO2 fingerprint is the limiting motif and could be considered a super-hallmark of type I BVMOs (Fig. 1A) (https://smartbioinf.github.io/BVMO_type1_Tomas_et_al_2021/). In other words, the observation of BVMO2 confirms the presence of the other motifs and thus certifies the existence of a BVMO enzyme. In addition, our analysis showed that M. tuberculosis counts 6, M. vaccae counts 12, and Rhodococcus jostii counts 8 putative type I BVMO candidates, whereas E. coli, B. subtilis, and C. glutamicum genomes have none (Fig. 1A). In M. tuberculosis, the six BVMOs, identified by sequence mining and confirmed by structural assessment, are the previously characterized BVMOs EthA and MymA, plus additional Rv1393c, Rv0565c, Rv0892, and Rv3049c candidates (Fig. 1B). Interestingly, EthA is the most largely conserved BVMO in the set of selected actinobacteria, with its presence in all genomes that contain BVMOs, and especially in M. leprae, which contains only one BVMO (Fig. 1A). Also, out of the four additional M. tuberculosis BVMOs, the annotated monooxygenase Rv0565c has an activity recently shown to confer susceptibility to ethionamide (ETH) and its analog prothionamide (PTH) (6). Rv0892, Rv1393c, and Rv3049c are annotated in UniProt as probable monooxygenases. Accordingly, the phylogenetic tree highlights that EthA, MymA, and Rv0565c cluster on the same branch, while Rv0892 is located separately, on another clade, and Rv1393c and Rv3049c are positioned even further away on more remote branches (Fig. 1B). Also, among the 6 BVMOs, EthA, MymA, Rv0565c and Rv0892 display (i) a probability of 100% to share structural homology with TmCHMO (*Thermocrispum municipale* CycloHexanone MonoOxygenase a type I BVMO), (ii) an e-value below 1.e-15, and (iii) an alignment core of minimum 300 residues (Fig. 1C). Reversely, Rv3520c, primarily pulled out because it displays two Rossmann motifs, was not selected *in fine* because it contains none of the BVMO motifs (see Fig. S1A in the supplemental material). Nevertheless, to challenge it as a true negative, it was kept for later experimental validation (Fig. S1B). For all those reasons, we focused our *in silico* analysis on EthA, MymA, Rv0892, and Rv0565c proteins.

10.1128/mSphere.00482-21.1 FIG S1 (A) *In silico* screening for BVMO signature motifs in the six M. tuberculosis BVMOs. Rv3520c is a monooxygenase displaying only two Rossmann motifs and no BVMO1 or -2, serving as negative control. (B) Assessment of BVMO activity in purified Rv3520c protein and comparison to the control BVMO (commercial cyclohexanone monooxygenase [CHMO]). The assay consisted in monitoring NADH oxidation (expressed as slopes) in the presence of cyclohexanone (CH) and ethyl 3-oxohexanoate (Et3-ox). Download FIG S1, TIF file, 0.2 MB. Copyright © 2022 Tomas et al. 2022 Tomas et al. https://creativecommons.org/licenses/by/4.0/ This content is distributed under the terms of the Creative Commons Attribution 4.0 International license.

### Homology modeling and substrate profiling of M. tuberculosis BVMOs.

**FIG 2:**
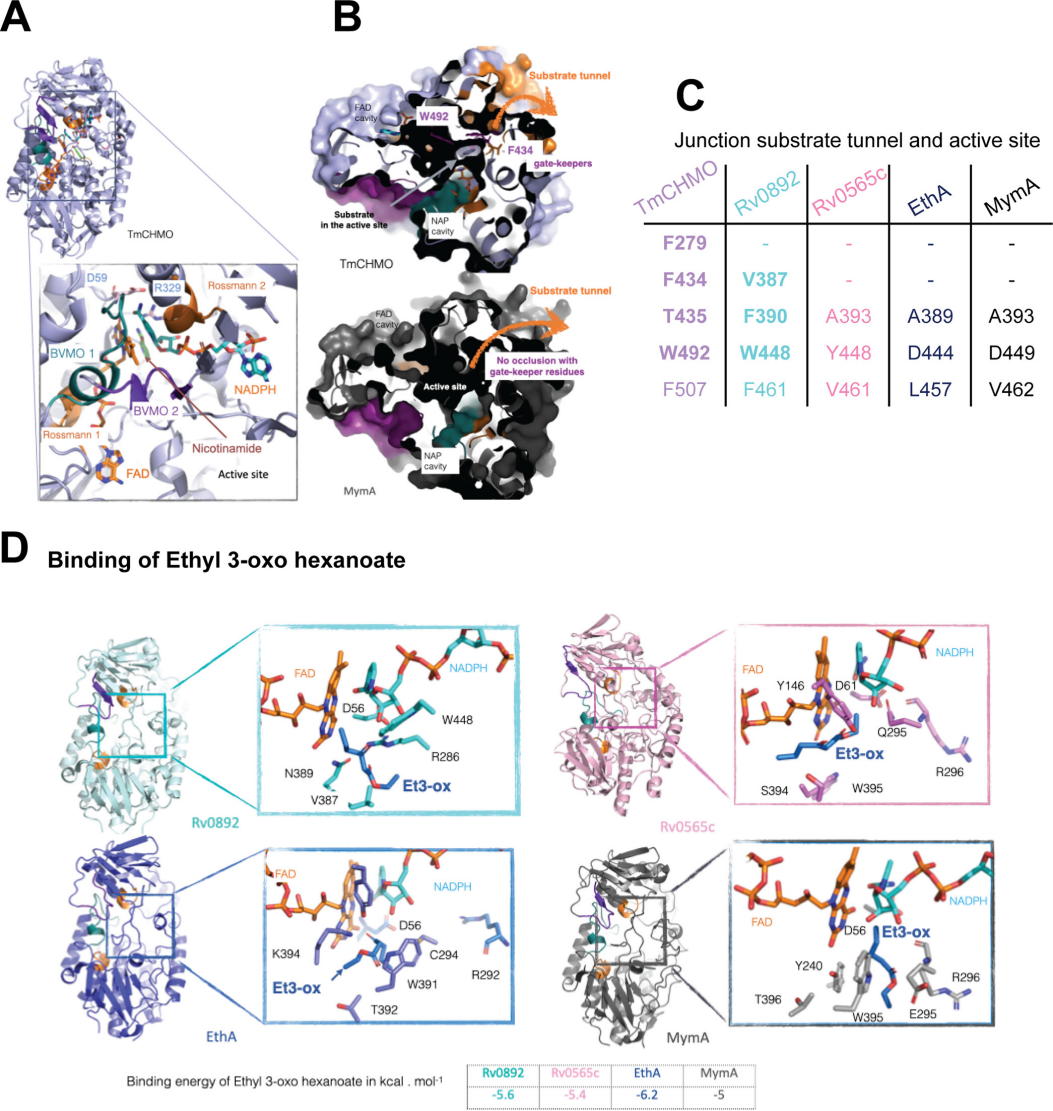
Homology modeling of M. tuberculosis BVMOs. (A) Mapping of BVMO fingerprints onto the 3D structure of *Tm*CHMO. The fingerprints have the same color code as in Fig. 1C, with Rossmann 1 and 2 colored in orange, and BVMO1 and BVMO2 colored in deep teal and purple, respectively. Inserted below is a close view of the active site, with the binding of FAD and NADPH cofactors. The catalytic residues D59 and R329 are shown as sticks. (B) Surface cross section of *Tm*CHMO and MymA. In the former, the accessibility of ligands could be sterically hampered by a restraint at the junction between the substrate tunnel and the catalytic site, possibly due to gatekeeper residues composed of F279, F434, and W492. Reversely, in MymA, the active site is openly accessible directly through the substrate tunnel as no residues hamper the junction between the substrate tunnel and the catalytic site. (C) Table with the listing of residues that form the junction between the substrate tunnel and the active site for the four modeled enzymes. From Rv0892 to MymA, the residues are progressively fewer and smaller, so one can hypothesize that atomic hindrance could play for substrate selectivity through a steric gradient from restricted for Rv0892 to relaxed for MymA. (D) Homology modeling of Rv0892, MymA, EthA, and Rv0565c. Inserted are close views of the docking of ethyl 3-oxohexanoate with details of residues involved in binding as well as a table of binding energy in kilocalories · mole^−1^.

In order to assess how EthA, MymA, Rv0565c, and Rv0892 fold into a type I BVMO structure, and how they accommodate NADPH and FAD cofactors, we built a homology model for each protein, with *Tm*CHMO as the template (PDB ID 5M10) (Fig. 2A). The catalytic dyad is formed by the strictly conserved aspartate residue, which belongs to the BVMO1 motif, and by a conserved arginine residue that does not belong to any conserved motif (red stars in Fig. 1C). Expectedly, the signature sequence motifs arrange similarly in 3D, bind cofactors FAD and NADPH, and participate to form the active site (Fig. 2B). As they are all located on the N-terminal side of the structure, this part of the active site is conserved. Reversely, the C-terminal part of the protein concentrates the differences in sequences, as overall similarity compared to *Tm*CHMO decreases from 40% to 31%, 30%, and 21% for Rv0892, Rv0565c, EthA, and MymA, respectively. Among the main discrepancies which can be highlighted, one can note that F279, F434, and T435, highlighted as purple stars in Fig. 1C, are located in insertions in *Tm*CHMO and that W492 is conserved only in Rv0892 with W448 (Fig. 2B and C). Interestingly, these substitutions locate at the junction between the substrate tunnel and the active site (11). Indeed, the *Tm*CHMO junction site is composed of residues F279, F434, T435, W492, and F507, which could be responsible for a steric barrier and could act as gatekeepers to accept substrates or not. With V387, F390, W448, and F461, Rv0892 keeps almost all large and hydrophobic residues at these positions (Fig. 2B and C). Conversely, these residues are much less conserved in Rv0565c, EthA, and MymA, and when substituted in three out of five residues, they are replaced by smaller or even polar residues (Fig. 2C). Their substitutions into smaller residues or even their absence could result in a distinct substrate selectivity profile. Thus, one can hypothesize that these discrepancies could act on substrate acceptance and contribute to distinct binding profiles. Altogether, these data led to the hypothesis that the four M. tuberculosis monooxygenases could share a similar reaction mechanism, while they likely differ in their substrate selectivity. To challenge that assumption, we performed *in silico* docking and then *in vitro* enzymatic studies.

To establish the substrate profiling and evaluate the substrate preferences of the four enzymes, four ligands mimicking either cyclic or linear substrates were docked in each active site, and their interaction energy was computed accordingly. The best pose, among the 10 runs performed for each pair of protein and ligand and with respect to the interaction energy, was kept. As shown for the ethyl 3-oxohexanoate (Fig. 2D) and more largely for the panel of ligands, the affinity is comparable for any combination of enzyme and ligand, ranging from −4.5 to −6.2 kcal · mol^−1^ (Table S2). From our point of view, such discrepancy in interaction energy is not significant enough to assess a strict preference, whether it concerns enzyme or substrate. It is important to acknowledge that a docking is a pose of a molecule once captured within the cavity pocket and not its behavior along the diffusion pathway. This point is particularly relevant when considering not only the binding of the small molecule but its accessibility to reach the reactive center, as well as its capacity to trigger enzyme motion and conformational change to accommodate a substrate. Indeed, the discrepancies in sequences are mapped in the structure partly within the close vicinity of the so-called N-terminal hinge linker (I151-D153 *Tm*CHMO numbering) but mostly within the C-terminal domain (Fig. 1C). The first segment, along with the second linker hinge, which is more conserved, A379-G381 (*Tm*CHMO numbering), could be involved in domain rotation, upon ligand binding. This has already been described for a similar topology domain of thioreductases (12). Moreover, the second series of substitutions impacts mostly residues involved in the substrate tunnel. Particularly, they concentrate at the junction formed by the end of the tunnel and the entrance to the active site; those residues are highlighted as orange spots in Fig. 1C and depicted as orange surface in Fig. 2B (13).

10.1128/mSphere.00482-21.7 TABLE S2 Docking results with interaction energy in kilocalories · mole^−1^. Cyclohexanone (CH), thioanisole (ThioA), ethyl 3-oxohexanoate (Et3-ox), and 2-octanone (2-oct). Download Table S2, DOCX file, 0.01 MB. Copyright © 2022 Tomas et al. 2022 Tomas et al. https://creativecommons.org/licenses/by/4.0/ This content is distributed under the terms of the Creative Commons Attribution 4.0 International license.

Taken together, homology modeling and substrate docking validate that the four proteins should be BVMO enzymes. They possess the 3D fold typical of this class of enzymes, accommodate both cofactors and ligand, and eventually prime the very first shell of catalytic residues as ready for oxidation. In addition, *in silico* characterization addresses the point that differences on sequences could impact NADPH domain motion and substrate acceptance; especially, discrepancies along the substrate tunnel could tune the substrate profiling. It also emphasizes that Rv0892 is closer to *Tm*CHMO with respect to gatekeeper residues and hinge N-terminal linker. From Rv0892 to MymA, the residues are progressively fewer and smaller, so one can hypothesize that substrate selectivity, based on steric bulk from restricted for Rv0892 to relaxed for MymA, could tune the accessibility from outside. To conclude, Rv0892 and *Tm*CHMO should partake in substrate profiling, sensitively distinct from Rv0565c, EthA, and MymA, which could overlap substrate profile similarity, due to closer sequences and comparable 3D arrangement. Therefore, we expect that Rv0892 will catalyze oxidation of similar substrates as those of *Tm*CHMO, while Rv0565c, EthA, and MymA could line up similar substrate profiling behavior, with subtle specificity due to residue substitutions (i.e., highlighted as blue arrows, solid orange circles, and purple stars in Fig. 1C).

### Purification and substrate selectivity profiles of M. tuberculosis BVMOs.

In order to gain knowledge into the possible function and putative distinctive substrate selectivity of MymA, EthA, Rv0565c, and Rv0892, we overexpressed and optimized purification of the four proteins. Except for the reported purification of EthA (14, 15), previous reports in the literature described either assaying activity using crude extracts overexpressing M. tuberculosis BVMOs (16) or unsuccessful attempts to purify the proteins, likely due to their low solubility in classical expression systems (6).

We cloned the four corresponding genes into E. coli pET expression plasmids (Table S1) and performed multiple expression and purification optimization assays. All attempts to express Rv0565c and Rv0892 under soluble forms were unproductive whereas we succeeded in purifying MymA*_coli_* and EthA*_coli_* proteins to homogeneity and with relatively good yields (Fig. S2A).

10.1128/mSphere.00482-21.2 FIG S2 Purification of M. tuberculosis BVMO. The proteins were expressed in E. coli (A) or in M. smegmatis (B) and purified as described in the text. Purified fractions were analyzed on a 4 to 20% stain-free precast SDS-PAGE gel (Mini-Protean TGX; Bio-Rad) and stained with InstantBlue (Expedeon) or revealed by fluorescence imaging (GelDoc; Bio-Rad). Anti-His immunodetection confirmed the presence of BVMO proteins in elution fractions. The table indicates molecular weights and purification yields from one representative purification. Download FIG S2, TIF file, 0.4 MB. Copyright © 2022 Tomas et al. 2022 Tomas et al. https://creativecommons.org/licenses/by/4.0/ This content is distributed under the terms of the Creative Commons Attribution 4.0 International license.

10.1128/mSphere.00482-21.6 TABLE S1 Cloning primers used for heterologous expression of recombinant 6×His-tagged M. tuberculosis BVMOs in M. smegmatis (pLD1 and pVV2 vectors) and E. coli (pET28a vector). Download Table S1, DOCX file, 0.01 MB. Copyright © 2022 Tomas et al. 2022 Tomas et al. https://creativecommons.org/licenses/by/4.0/ This content is distributed under the terms of the Creative Commons Attribution 4.0 International license.

To comparatively dissect the substrate preferences of all selected BVMOs, we therefore implemented an optimized expression and purification workflow in the mycobacterial host strain M. smegmatis, already successfully used as an efficient surrogate host to express M. tuberculosis proteins (17). Indeed, expression of the four recombinant 6×His-tagged BVMOs in M. smegmatis allowed their significant enrichment, as evidenced by stain-free analysis and immunodetection (Fig. S2B).

**FIG 3:**
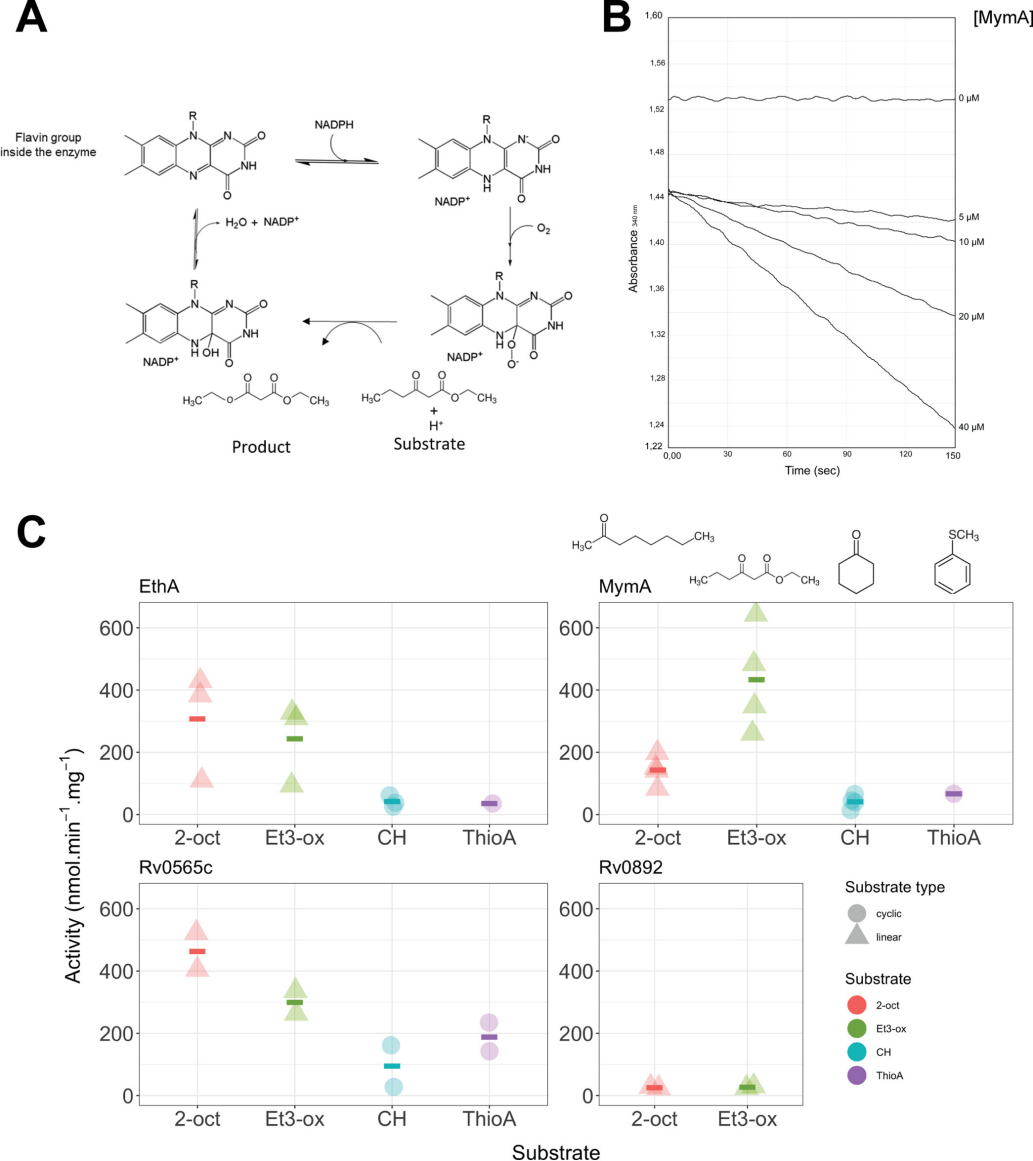
*In vitro* BVMO assay and substrate profiling. (A) Catalytic mechanism of BVMO with the different oxidation states of the FAD. (B) Kinetics of NADPH oxidation at 340 nm in the presence of MymA. The reactions were carried out for 4 min at 25°C with 5 mM ethyl 3-oxohexanoate (Et3-ox) as the substrate and started by addition of increasing concentrations of MymA, as described under Materials and Methods. (C) Activity of EthA, MymA, Rv0565c, and Rv0892, in the presence of various substrates: cyclohexanone (CH), thioanisole (ThioA), ethyl 3-oxohexanoate (Et3-ox), and 2-octanone (2-oct). Enzyme concentration in the assay was 10 to 40 μg/mL, the substrates were used at 5 mM (1 mM for thioanisole) and NADPH was used at 0.2 mM. Activity rates correspond to nanomoles of NADPH consumed per minute per milligram of protein for each BVMO. Circles represent the mean values for at least two protein extraction replicates for a given independent experiment.

Despite the low purification yields in this expression system, we were able to investigate the *in vitro* activity of the four proteins in the presence of selected aliphatic and cyclic substrates. The assay designed to assess the BVMO activity consisted in following the NADPH oxidation coupled to monooxygenation of the substrate, as previously described (18) (Fig. 3A). Validation of the spectrophotometric assay was performed using commercial CHMO and the non-BVMO Rv3520c as positive and negative controls, respectively (Fig. S1B). Notably, these results reinforce the strength of our *in silico* strategy to distinguish between BVMO and non-BV monooxygenases (Fig. S1A).

All purified BVMOs were assessed for their activity, by checking that the measured NADPH oxidation is dependent upon—and proportional to—enzyme concentration (protein dose-response curve), as exemplified by MymA (Fig. 3B). The four purified proteins were then assayed under the same experimental conditions, in biological and technical replicates in the presence of the molecules 2-octanone (2-oct), thioanisole (ThioA), ethyl 3-oxohexanoate (Et3-ox), and cyclohexanone (CH).

All four BVMOs display a Baeyer-Villiger oxygenase activity and similar substrate profiles, with 2-oct and Et3-ox being the most effective substrates. A similar experimental substrate profiling can be noted for Rv0565c, EthA, and MymA with limited to marked increasing oxidation from cyclic to linear substrate (Fig. 3C). Particularly, Rv0565c and EthA are efficient in oxidizing 2-octanone while MymA is very active on ethyl 3-oxohexanoate. Rv0892 exhibits markedly low catalytic rates on the tested substrates (specific activities, 27 nmol.min^−1^.mg^−1^ for Et3-ox and 23 nmol.min^−1^.mg^−1^ for 2-oct); nevertheless, its activity shows that it is a BVMO enzyme (Fig. S3).

10.1128/mSphere.00482-21.3 FIG S3 Activity of Rv0892 in the presence of the linear substrates. Protein response curve of Rv0892 at 20 and 40 μg/mL. The assay consisted in monitoring NADH oxidation at 340 nm, and reactions were carried out for 4 min at 25°C with 0.2 mM NADPH and a 5 mM concentration of the substrate ethyl 3-oxohexanoate (Et3-ox) or 2-octanone (2-oct) and started with addition of enzyme. Download FIG S3, TIF file, 0.04 MB. Copyright © 2022 Tomas et al. 2022 Tomas et al. https://creativecommons.org/licenses/by/4.0/ This content is distributed under the terms of the Creative Commons Attribution 4.0 International license.

EthA and Rv0565c seem to prefer 2-oct to Et3-ox (Fig. 3C) over the cyclic substrates; however, analysis of variance (ANOVA) and *post hoc* tests could not provide sound statistical proof for this difference (Fig. S4). Most notably, though, a robust proof of the opposite activity pattern was produced for MymA, which presents significantly higher activity with Et3-ox compared to 2-oct.

10.1128/mSphere.00482-21.4 FIG S4 Ninety-five percent confidence intervals (CI) obtained by general linear hypothesis *post hoc* test (glht) on chosen contrasts taken from the interaction effect between substrates and BVMO candidates (described in the legend to Fig. 3C). This interaction effect was in itself significant (ANOVA, *P* value = 0.017). For a given enzyme, the difference in activity between the linear (i.e., Et3-ox versus 2-oct) or cyclic (i.e., ThioA versus CH) substrates was tested. Download FIG S4, TIF file, 0.1 MB. Copyright © 2022 Tomas et al. 2022 Tomas et al. https://creativecommons.org/licenses/by/4.0/ This content is distributed under the terms of the Creative Commons Attribution 4.0 International license.

### Assay adaptation and enzymatic characterization of MymA.

In order to gain further insights into MymA enzymatic properties with regard to its distinctive substrate profile and given the increasing interest in this anti-TB prodrug activator, we miniaturized the BVMO assay using purified MymA*_coli_* enzyme for which we obtained pure protein and high-purification yields (1.7 mg/L culture [Fig. S1A]). We first checked its activity compared to that of the protein produced in M. smegmatis. Indeed, MymA*_coli_* was active in the presence of Et3-ox (Fig. S5A). We then set up a microplate assay for NADPH oxidation to follow the BVMO activity and showed that comparable specific activities for MymA*_coli_* were obtained using either a cuvette or a 96-well plate format (Fig. S5B).

10.1128/mSphere.00482-21.5 FIG S5 Activity assessment of MymA*_coli_* and adaptation of BVMO assay to microplate. (A) Protein curve analysis of MymA expressed in E. coli (MymA*_coli_*) and compared to the MymA expressed in M. smegmatis. (B) Protein curve analysis of MymA expressed in E. coli in assays realized in a cuvette and in 96-well microplates. Download FIG S5, TIF file, 0.1 MB. Copyright © 2022 Tomas et al. 2022 Tomas et al. https://creativecommons.org/licenses/by/4.0/ This content is distributed under the terms of the Creative Commons Attribution 4.0 International license.

**FIG 4:**
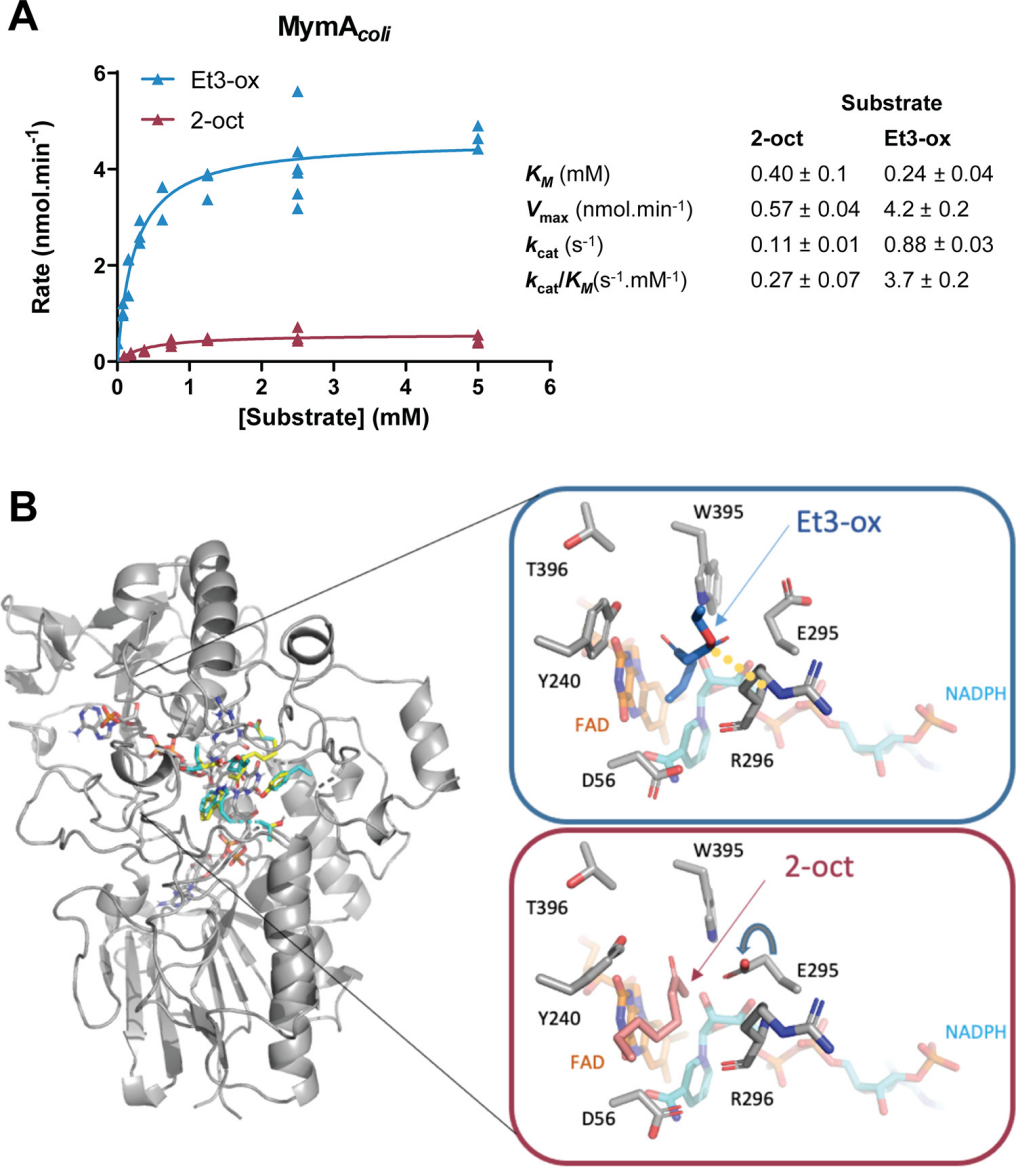
MymA substrate selectivity. (A) Steady-state kinetics of MymA produced in E. coli (MymA*_coli_*) with ethyl 3-oxohexanoate (Et3-ox, blue triangles) and 2-octanone (2-oct, red triangles). Activities were determined at 25°C by following the NADPH consumption in 96-well microplates, in a 250-μL volume. Reaction mixtures contained 0.5 mM NADPH and increasing concentrations of the substrate (0 to 5 mM) and were started by addition of MymA*_coli_* at 20 μg/mL. Data were fitted to the Michaelis-Menten equation, and kinetic parameters for Et3-ox and 2-oct were determined using GraphPad Prism 5.04. (B) Docking of Et3-ox and 2-oct on MymA, in blue and salmon, respectively. Inset is a closeup view of the binding. The rotation of E295 is shown by a gray arrow, and the polar bond between the ligand and R296 is highlighted by a yellow dashed line.

Using the microplate assay and MymA*_coli_*, we therefore determined the catalytic constants for Et3-ox and 2-oct (Fig. 4A). The steady-state apparent parameters indicate higher catalytic constants (*k*_cat_) for Et3-ox (0.88 ± 0.03 s^−1^) compared to 2-oct (0.11 ± 0.01 s^−1^) and catalytic efficiency (*k*_cat_/*K_M_*), while the *K_M_* (Michaelis constant) values are marginally comparable for the two substrates (0.24 ± 0.04 mM and 0.4 ± 0.1 mM, respectively). The measured kinetic parameters are on the same order as those obtained for other BVMO-substrate pairs previously characterized (11, 18). This could correlate with docking analysis which shows that Et3-ox and 2-oct ligands engage similar binding energy with MymA. However, the best poses for 2-oct and Et3-ox evidence a difference in the side chain rotamer of E295, located within the first catalytic shell of protein residues. Such rotation of E295 could favor a polar interaction of Et3-ox both with the backbone of the catalytic R296 and with the side chain of W395 (Fig. 4B).

## DISCUSSION

In this work, we implemented an original bioinformatic pipeline, based on sequence mining and structural homology detection, not only to identify but also to visualize type I BVMOs. As a first main result, our bioinformatic pipeline was experimentally confirmed as accurate in identifying type I BVMOs in a series of genomes. It also evidenced that cooccurrence of two Rossmann motifs, plus BVMO1 and BVMO2 fingerprints, is required to form the hallmark characterizing type I BVMOs. Markedly, our bioinformatic analysis highlights that when BVMO2 is present, all other specific motifs are also present, which suggests that BVMO2 could define a “super” motif to screen for BVMO in entire genomes. Interestingly, this motif can eventually be degenerated (data not shown). Last, while the BVMO2 fingerprint is not strictly part of the active site, it belongs to the linker segment that connects the FAD-binding domain to the NADPH-binding domain of these monooxygenases (19). In line with that, Rv3520c, which harbors only two Rossmann motifs, was shown to be strictly inactive.

As a second result, we found BVMOs in all but one of the screened genomes of *Mycobacteriales*, which is C. glutamicum. The number of identified BVMOs present within these genomes varied from one, in M. leprae, which is EthA, to up to 12 in M. vaccae. For M. leprae, this is fully consistent with the work of Fraaije et al. (18). Also, we counted no fewer than 8 to 12 putative BVMOs for *M. phlei*, M. vaccae, *R. jostii*, or *N. farcinica*. Those bacteria are known to thrive in a broad range of environments, including soil, water, and eukaryotic cells. Also, they have large genomes. This broad distribution of BVMOs is likely attributable to the diversity of carbon sources available for these organisms. As BVMOs catalyze key reactions in metabolic pathways, the primary metabolism of atypical carbon sources could confer an adaptative behavior and an evolutionary advantage (7). These enzymes can adapt to a wide range of ketones as substrates to oxidize them into esters or lactones (7). Despite this physiologic redundancy, the majority of newly identified potential BVMOs remain to be formally functionally annotated and biochemically characterized in future studies.

We then focused on the human pathogen M. tuberculosis, which encodes 6 BVMOs as confirmed in our analysis. Earlier studies have demonstrated that M. tuberculosis uses two of these BVMOs, EthA and MymA, to activate ETH, highlighting their importance for the pharmacological mechanism of this class of TB drugs (3–5, 20, 21). Later it was shown that some other proteins, including Rv0565c, can also activate this drug (6). Our current analysis suggested that monooxygenase-annotated gene product Rv0565c represents a BVMO with structure and function similar to EthA and MymA, while Rv0892 could be closer to a regular cyclohexanone BVMO. We followed up analysis on testing this hypothesis. Our homology modeling revealed that all four enzymes are structural homologues, with local mutations mapped mainly within the substrate tunnel, likely impacting substrate accessibility rather than expected binding preference. Formerly, the substrate tunnel was suspected to select and drive ligands to the active center and expel products once oxidized. We docked a series of putative substrates (cyclic and aliphatic molecules) into homology models and observed that indeed all four enzymes share a similar binding behavior with respect to their interaction energy with ligands. Nevertheless, we could highlight some insertions or substitutions along the so-called substrate tunnel and at the entrance of the active site that could impact the destiny of the ligands. We also identified subtle mutations at the first hinge linker that is known to contact both BVMO1 and -2 motifs. This double-hinge β-sheet linker is conserved in Baeyer-Villiger monooxygenases, where it is suspected to be involved in domain motion. Possibly, substitution could act on NADPH domain motion, but at this stage, this assumption would need to be better documented. From *in silico* analysis and comparison with *Tm*CHMO, gatekeeper residues are more conserved in Rv0892 than in Rv0565c, EthA, and MymA. Thus, we hypothesize that Rv0892 could share a compact cavity similar to *Tm*CHMO and distinct from Rv0565c, EthA, and MymA; all three would gather in a similar topology (13, 22). This is relevant with respect to the phylogeny tree. To challenge this hypothesis, we further performed *in vitro* assays. They confirmed that Rv0892 behaves differently from Rv0565c, EthA, and MymA, which exhibit comparable enzyme activities and substrate specificities. Rv0565c, EthA, and MymA share *in vitro* a similar substrate profile, with respect to their tropism for the linear substrates 2-oct and Et3-ox. Markedly, MymA displays a distinct effective behavior when the linear substrates are considered. Reversely, Rv0892 is much less active on those substrates. In-depth investigation of Rv0892 behavior toward the cyclic substrate was hampered by very low protein yields and extremely low substrate conversion rates under assay conditions. Future purification and assay optimizations will be required to assess the ability of Rv0892 to oxidize CH and ThioA. Altogether, this work confirms that Rv0565c, EthA, and MymA are M. tuberculosis BVMOs and identifies Rv0892 as a new BVMO, with a likely distinct substrate selectivity that remains to be ascertained. Still, for M. tuberculosis we need to correlate BVMO substrate profiling with their physiological relevance within the cell, which is beyond the sole capacity to activate prodrugs. Open questions remain as to the versatile role of these M. tuberculosis BVMOs and their possible involvement in lipid metabolism (9).

Given that BVMOs are able to oxidize a broad spectrum of substrates, they likely exhibit some physiological redundancy. Therefore, we suggest that the presence of multiple BVMOs would allow an organism to readily adapt to a wide range of carbon sources. From this perspective, it is important to pursue the efforts and expand our knowledge of BVMO function with the ultimate goal of developing strategies for controlling and exploiting this class of enzymes in the context of anti-TB drug development.

## MATERIALS AND METHODS

### Bioinformatic analysis.

The genomes of mycobacteria, from M. tuberculosis, M. leprae, M. smegmatis, M. bovis, M. marinum, *M. phlei*, and M. vaccae, and the genomes of corynebacteria, from C. glutamicum, *N. farcinica*, *R. jostii*, *M. gilvum*, and *H. subflava*, were selected and downloaded from the NCBI site. The references Gram-positive Bacillus subtilis and Gram-negative Escherichia coli were also included. Every sequence of each genome was screened with the EMBOSS FuzzPro tool, to search for sequence motifs that display the hallmarks for type I BVMOs (23, 24). Such a sequence pattern contains two GxGx_2_[GA] motifs, so-called Rossmann ones, a [GA]GxWx_4_[FY]P[GM]x_3_D motif named here BVMO1, and a FxGx_3_Hx_3_W[PD] motif named here BVMO2, where the residues inserted in the rackets show the possible substitutions. With respect to the BVMO type I enzymatic signature, sequences that strictly associate fingerprints in the following order were kept: Rossmann motif-BVMO1-BVMO2-Rossmann motif. A dedicated bioinformatic pipeline was developed to identify readily all type I BVMOs within entire genomes. The pipeline, implemented to deliver an interactive comparison among a series of genomes, is available on GitHub (https://github.com/SmartBioInf/COSP). A phylogenomic analysis was performed on selected sequences with the FastME/OneClick workflow of NGphylogeny software (https://ngphylogeny.fr/) (25), and the resulting tree was visualized using iTOL (Interactive Tree Of Life; https://itol.embl.de) (26). Then, the sequences were computed with HHpred, using the default parameters and the structural database PDB_mmCIF70, to detect their structural homologues and assess if they all display the same 3D fold (27). In M. tuberculosis, the sequences that (i) retain pattern detection; (ii) show 100% probability of being structurally homologous to the template, cyclohexanone BVMO from Thermocrispum municipale (*Tm*CHMO) chosen upon HHpred; and (iii) distribute evenly on the phylogeny tree were selected for *in silico* and then *in vitro* characterization. Namely, these are Rv0892, Rv0565c, Rv3083 (MymA), and Rv3854c (EthA). For the sake of clarity, Rv3083 and Rv3854c are named here MymA and EthA, respectively.

### Homology modeling of full-length Rv0565c, Rv0892, MymA, and EthA.

Rv0892, Rv0565c, MymA, and EthA were homology modeled using *Tm*CHMO as the 3D template because it is one of the closest structural homologues identified by HHpred (27). Also, *Tm*CHMO is in complex with FAD, NADPH, and nicotinamide (5M10 PDB ID) (22). The model-building software Modeller was chosen (version 9.18) (28) starting from a multiple alignment of the four proteins plus the reference template, using the ClustalW algorithm for its accurate precision (with default parameters) (29) and subsequently using ESPript for its capacity to insert the secondary structure elements of the template (30). For each protein, 100 homology models were generated, the final structure that associates the lowest values of Modeller function and DOPE score was selected, and the stereochemistry was checked using MolProbity (31). Finally, the cofactors were added and each complex was minimized to relax local probable steric clashes, using Gromacs Charmm36 forcefield with the steepest-descent algorithm (32).

### Docking of ligands.

The molecule of nicotinamide was redocked in the binding pocket of *Tm*CHMO, to check if the protocol docks it similarly to its position in the crystal structure. As its pose was confirmed, it served as a positive control, and then the protocol was approved for subsequent docking of ligands in the binding sites of the homology-modeled Rv0892, Rv0565c, MymA, and EthA proteins. AutoDock tools were used, with a grid box centered on the crystal position of the nicotinamide and the genetic algorithm of Lamarck (33). The side chains of residues located within 4 Å from the nicotinamide were allowed to be flexible so that rotamer transition could be allowed. With anticipation, the grid box was sized to accommodate the largest ligand of the series, which was composed of cyclohexanone, thioanisole, 2-octanone, and ethyl 3-oxohexanoate. When proper, their dihedral angles were set free to rotate. Subsequently, each ligand was docked in the binding site of every BVMO protein, using similar docking parameters, so that resulting binding energies could be compared. For each ligand, the complex showing the best affinity between ligand and protein, i.e., the lowest binding energy computed in kilocalories · mole^−1^, was selected for molecular analysis. Finally, apo and holo models were visually inspected using PyMOL 2.0.7 (Schrödinger, LLC).

### Plasmid construction.

The *rv0892*, *rv3083* (*mymA*), and *rv3854c* (*ethA*) genes were cloned into the isopropyl-β-d-thiogalactopyranoside (IPTG)-inducible mycobacterial expression vector pLD1 (17) or pET28a using primers (see Table S1 in the supplemental material) designed to generate PCR product from the genomic DNA of M. tuberculosis strain H37Rv, containing the NotI/NdeI restriction sites for cloning into pLD1 or BamHI/HindIII for cloning into pET28a. The inserts were verified by sequencing (Eurofins Genomics, France). The *rv0565c* gene was cloned into the pVV2 expression vector (34) using the primers designed to generate a PCR product corresponding to the entire *rv0565c* gene from M. tuberculosis strain H37Rv and harboring NdeI/HindIII restriction sites enabling direct cloning into the pVV2 vector (Table S1). The insert was verified by sequencing (Microsynth, Switzerland).

### Bacterial strains and culture conditions.

For expression in M. smegmatis, the plasmids pLD1-*rv0892*, pLD1-*rv3083* (*mymA*), and pLD1-*rv3854c* (*ethA*) were electroporated into M. smegmatis mc^2^ 155 GroELΔC. Transformants were selected on 7H10-0.2% glycerol agar plates with hygromycin B (100 μg/mL). Starter cultures were grown for 30 h at 37°C under shaking and then diluted in 2 L of 7H9 medium (Difco) supplemented with glycerol (0.2%), tyloxapol (0.025%), and hygromycin B (100 μg/mL). The resulting cultures were incubated under shaking (180 rpm) at 37°C until reaching an optical density at 600 nm (OD_600_) of 0.6. BVMO overexpression was induced with IPTG (0.5 mM final concentration) (Euromedex, France). Cells were harvested 3 h after IPTG induction, pelleted by centrifugation, flash-frozen, and kept at −80°C.

For expression of Rv0565c, M. smegmatis GroEL1ΔC cells were transformed with pVV2-*rv0565c* and cells were stored with 25% glycerol. A 100-mL starter culture of LB medium containing 0.05% Tween 80 and 20 μg/mL kanamycin was inoculated at 1:100 from the glycerol stock and grown at 37°C under agitation until reaching an OD_600_ of ∼1.5. Two-liter cultures in LB media containing 20 μg/mL kanamycin inoculated with 20 mL of the starter culture were cultivated for 48 h at 30°C under agitation. Cells were harvested by centrifugation, and pellets were washed with 25 mM Tris-HCl, pH 7.5, 300 mM NaCl.

For expression in E. coli, One Shot BL21 Star(DE3) chemically competent cells were transformed with pET28a-*rv3083* or pET28a-*rv3854c* and grown in 500 mL of LB medium supplemented with 50 μg/mL kanamycin at a temperature of 37°C under agitation. Induction was performed at an OD_600_ of 0.6 by the addition of 50 μM IPTG for MymA or 250 μM for EthA. Cells were harvested after an overnight induction period at 16°C, pelleted by centrifugation, washed with phosphate-buffered saline (PBS) (Euromedex), and stored at −80°C.

### Purification of recombinant EthA and MymA (produced in E. coli).

Cell pellets (2 g, wet weight) were thawed and resuspended in 50 mL lysis buffer (50 mM HEPES, pH 7.0 for MymA or 50 mM sodium phosphate, pH 7.5, for EthA, both supplemented with 300 mM NaCl, 10 mM imidazole, 8% glycerol). One milligram/milliliter lysozyme, 0,1% Triton X-100, 1 mM AEBSF [4-(2-aminoethyl)benzenesulfonyl fluoride hydrochloride], and 1 mM dithiothreitol were also added in this lysis buffer. The suspension was lysed on ice with an Emulsiflex C5 homogenizer (Avestin) using 100,000-kPa pulses, and clarified lysate was obtained after centrifugation at 22,000 × *g* for 30 min. The clarified lysate was applied to a 1-mL HisTrap HP column (Cytiva) equilibrated with lysis buffer supplemented with 20 mM imidazole. Nonspecific bound proteins were eluted from the beads with lysis buffer supplemented with 90 mM imidazole. Proteins of interest were eluted with elution buffer (lysis buffer supplemented with 300 mM imidazole).

Eluted fractions were 10-fold concentrated using Vivaspin 20 (30-kDa cutoff; Cytiva), and 500-μL fractions were loaded at 0.5 mL/min on a Superose 6 Increase 10/300 GL (Cytiva) equilibrated with buffer (50 mM HEPES, pH 7.0 or 50 mM sodium phosphate, pH 7.5, 300 mM NaCl for MymA and EthA, respectively). The collected fractions were concentrated, and total protein concentration was determined using the bicinchoninic acid (BCA) protein assay kit (Sigma-Aldrich).

### Purification of recombinant EthA and MymA (produced in M. smegmatis).

Cell pellets (10 g, wet weight) were thawed and resuspended in 50 mL lysis buffer (50 mM sodium phosphate, pH 7.5, 300 mM NaCl, 8% glycerol) supplemented with 2 mM AEBSF. The suspension was lysed with a cell disruptor (One Shot model; Constant System Ltd., France) at 260,000 kPa. The clarified lysate obtained after centrifugation at 20,000 × *g* for 20 min was incubated with a volume of immobilized-metal affinity chromatography (IMAC) resin (Ni-NTA Agarose, Qiagen). After 2 h of incubation at 4°C, under agitation, the beads were recovered and washed with lysis buffer supplemented with 10 mM and then 60 mM imidazole. The fractions obtained upon elution with 300 mM imidazole were desalted on a PD10 Midi TrapG-25 column (Euromedex) and concentrated using a Vivaspin 20 (10-kDa cutoff; Sartorius).

### Purification of recombinant Rv0892 (produced in M. smegmatis).

Cell pellets (10 g, wet weight) were thawed and resuspended in 50 mL lysis buffer (50 mM sodium phosphate, pH 7.5, 300 mM NaCl, 8% glycerol) supplemented with 1 mM AEBSF. The suspension was lysed with a cell disruptor (One Shot model; Constant System Ltd., France) at 260,000 kPa. The clarified lysate obtained after centrifugation at 20,000 × *g* for 20 min was injected on a 1-mL HisTrap HP column (Cytiva) and then washed with lysis buffer supplemented with 90 mM imidazole. The fractions obtained upon elution with 300 mM imidazole were desalted on a PD10 Midi TrapG-25 column (Euromedex) and concentrated using a Vivaspin 20 (30-kDa cutoff; Cytiva).

### Purification of recombinant Rv0565c (produced in M. smegmatis).

Cell pellets (20 g, wet weight) were resuspended in 30 mL lysis buffer (25 mM Tris-HCl, pH 7.5, 300 mM NaCl) and disrupted by sonication on ice (60 s on, 90 s off, 15 times, 1-cm probe; Soniprep 150; Sanyo). The clarified lysate obtained after centrifugation at 10,000 × *g* for 10 min at 4°C was injected on a HisTrap Talon crude 1-mL (Cytiva) affinity column. The selected fractions obtained upon elution using a 5 to 500 mM imidazole gradient were concentrated using an Amicon Ultra filter (10-kDa cutoff), 4°C, 4,000 × *g*. The imidazole was removed by several washings using an Amicon Ultra filter. To obtain as much isolated protein as possible, the fraction of unbound proteins was subjected to the same isolation protocol.

For all purified BVMOs, total protein concentration was determined using a DS-11 spectrophotometer (DeNovix) or a BCA protein assay kit (Sigma-Aldrich). Proteins were separated on a 4 to 20% stain-free precast SDS-PAGE gel (Mini-Protean TGX; Bio-Rad) revealed by tryptophan fluorescence enhanced when exposed to UV light (GelDoc, stain-free imaging technology; Bio-Rad) and stained with InstantBlue (Expedeon). Quantification of protein bands on the gel was estimated by densitometry analysis using ImageLab (Bio-Rad).

### Immunoblotting.

Total cell lysates and eluted proteins were fractionated on a 4 to 20% stain-free precast SDS-PAGE gel (Mini-Protean TGX; Bio-Rad) and then transferred to a 0.4-μm nitrocellulose membrane using the Transblot Turbo semidry transfer method (Bio-Rad). Following the transfer, 6×His-tagged BVMOs were detected using mouse monoclonal anti-poly(His) antibodies (Sigma-Aldrich) diluted 1/5,000; horseradish peroxidase (HRP)-conjugated goat antibodies against mouse (Bio-Rad), diluted 1/5,000, were used as secondary antibodies. Detections were performed with the Amersham 383 ECL Prime Western blotting reagent kit (GE Healthcare). Immunoreactive bands were revealed and measured with the ChemiDoc imaging system (Bio-Rad).

### BVMO activity assay.

The assays were performed at 25°C with the UV mc^2^ spectrophotometer (SAFAS Monaco) in quartz cuvettes in a 450-μL reaction mixture (50 mM HEPES, pH 7.5, bovine serum albumin [BSA; 2 μM], NADPH [200 μM]). The activity was measured by monitoring the oxidation of NADPH at 340 nm (ε_340_ = 6,220 M^−1^ · cm^−1^) for 2 to 4 min. Cyclohexanone (CH, CAS no. 3249-68-1), 2-octanone (2-oct, CAS no. 111-13-7), ethyl 3-oxohexanoate (Et3-ox, CAS no. 3249-68-1), and thioanisole (ThioA, CAS no. 100-68-5) were provided by Sigma-Aldrich, prepared in ethanol at 60 mM, and used at 5 mM in the assay, except for thioanisole, which was prepared at 12 mM and used at 1 mM. Rv0565c, Rv0892, MymA, and EthA produced in M. smegmatis and purified as described above were used at 10 to 40 μg/mL. CHMO commercial enzyme was provided by Sigma-Aldrich and used at 0.05 U. Calculated activities represent nanomoles of NADPH consumed per minute per milligram of enzyme. Each experiment was performed at least in biological duplicates (independent cultures and protein purification batches) and at least in technical triplicates.

### Steady-state kinetics of MymA.

Kinetic parameters were determined using 96-well microplates (Nunc 96) in a CLARIOstar^Plus^ (BMG Labtech) plate reader. The reaction mixture (in a 250-μL final volume) contained 50 mM HEPES, pH 7.5, NaCl (50 mM), NADPH (500 μM), BSA (2 μM), 0 to 5 mM substrate, and purified MymA produced in E. coli at 20 μg/mL. Reactions were started by addition of enzyme and followed during 9 min (30 cycles, 22 flashes per well and cycle) at 340 nm and 25°C to follow consumption of NADPH.

### Statistical analysis.

Statistical analysis was carried out and subsequent figures were made under an R (v4.0.3) working environment and with RStudio interface (v1.2.5001) (35, 36). Base stats packages were used for standard procedures such as linear regression or ANOVA. Four-parameter log-logistic dose-response models were built and treated with the drc (v3.0.1) (37). Benjamini-Hochberg (BH) *P* value correction was duly implemented using the multcomp (v1.4.16) package (38, 39). The same package was used to produce general linear hypothesis *post hoc* tests. Data wrangling and visualization of results were handled with Tidyverse (v1.3.0) (40). R source codes are available upon request.
