# Supplementary figures and images for: Bioinformatic Mining and Structure-Activity Profiling of Baeyer-Villiger Monooxygenases from Mycobacterium tuberculosis
Source: mSphere. 2022 Mar 17;7(2):e00482-21. doi: 10.1128/msphere.00482-21 (PMC9044951; doi:10.1128/msphere.00482-21)

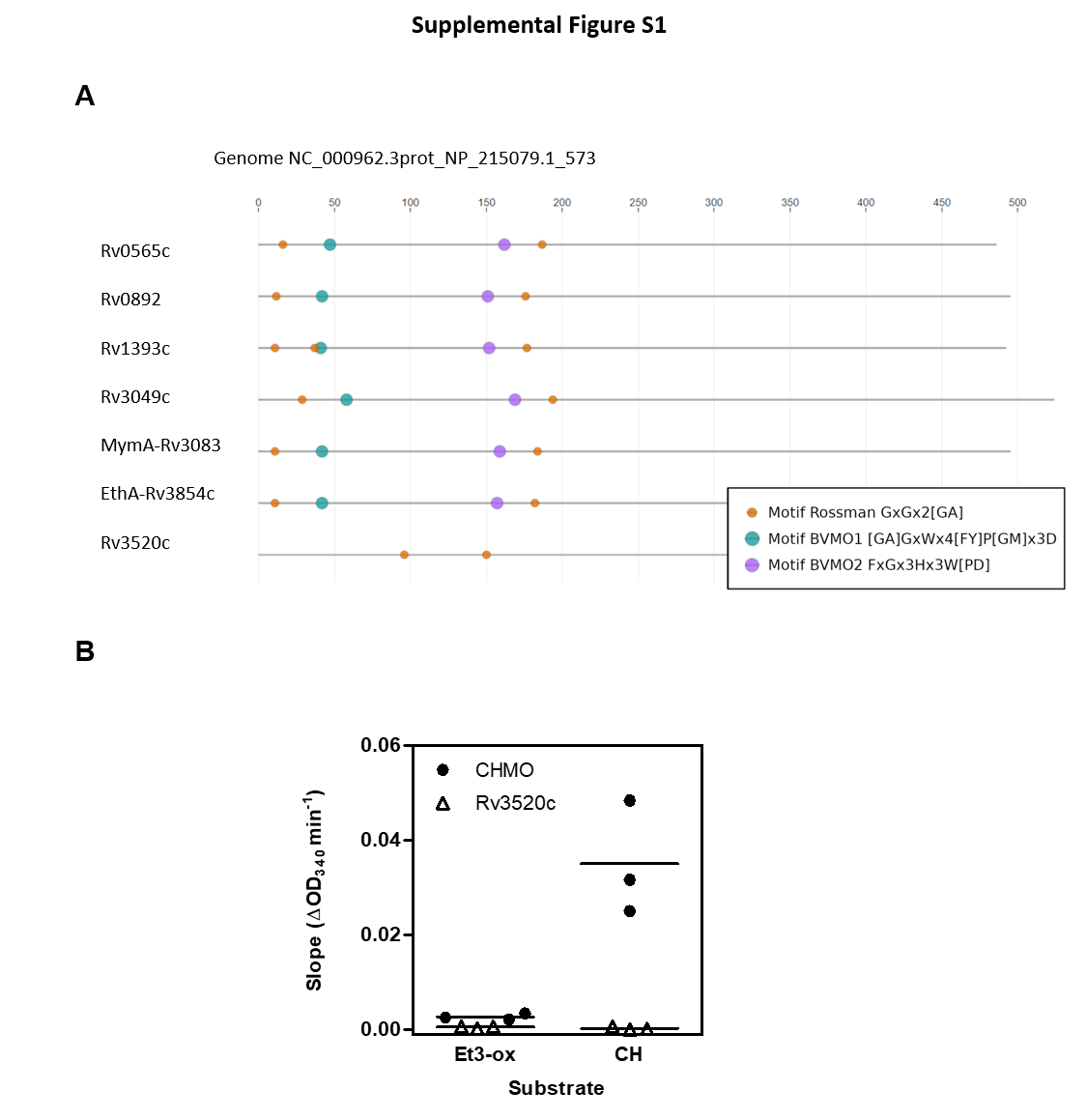

Supplement: FIG S1 [file msphere.00482-21-sf001.tif]

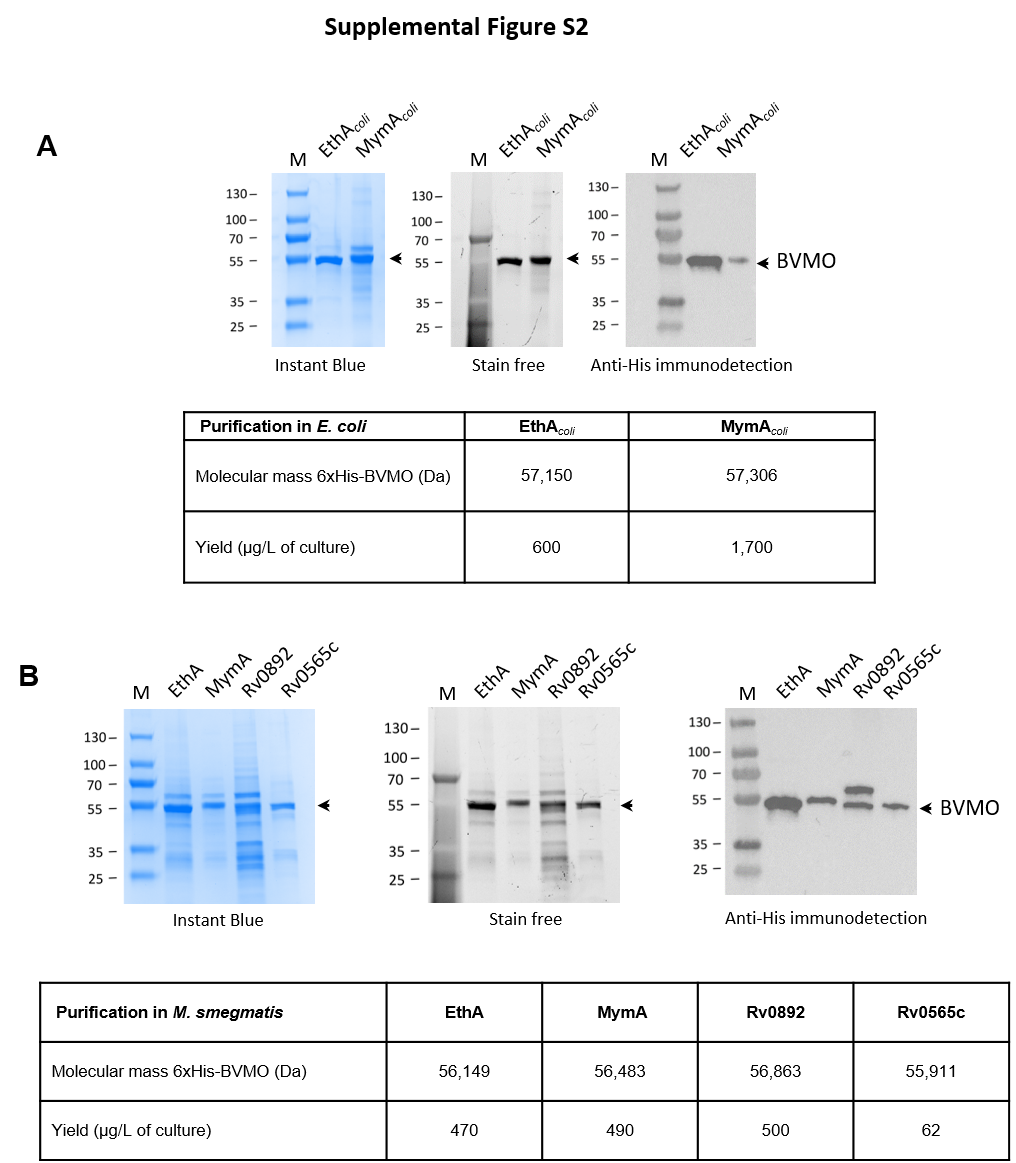

Supplement: FIG S2 [file msphere.00482-21-sf002.tif]

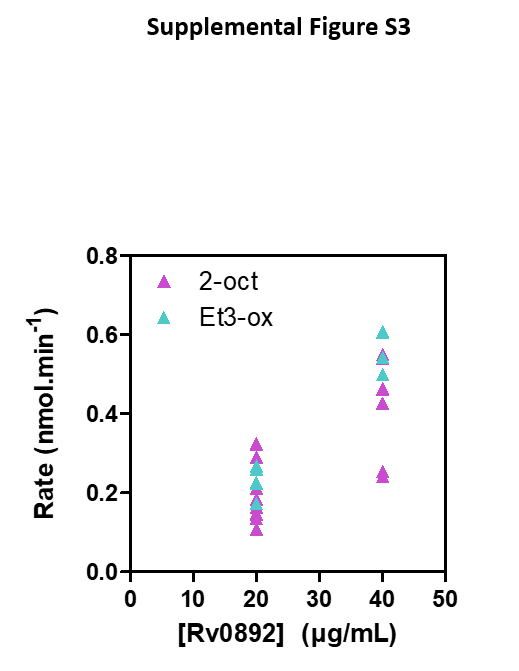

Supplement: FIG S3 [file msphere.00482-21-sf003.tif]

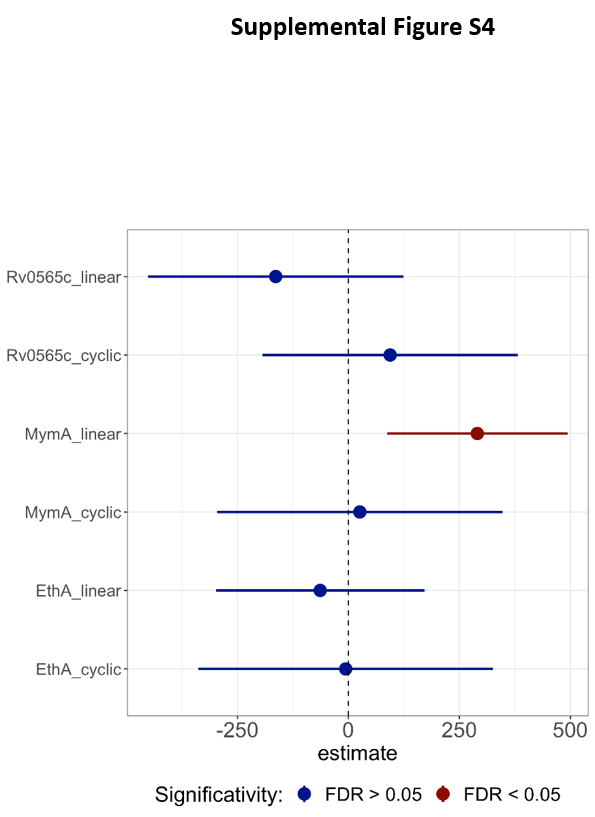

Supplement: FIG S4 [file msphere.00482-21-sf004.tif]

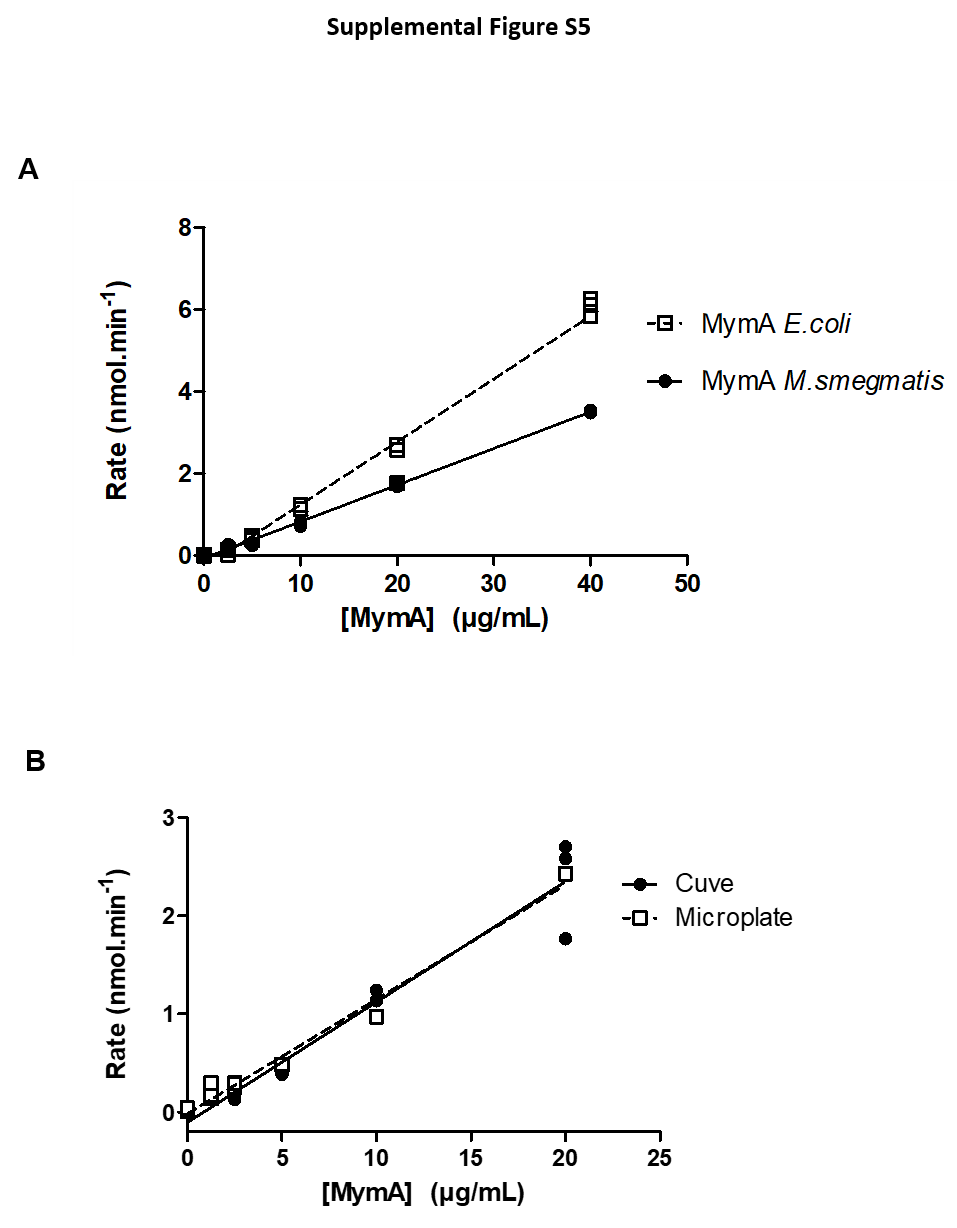

Supplement: FIG S5 [file msphere.00482-21-sf005.tif]
