# Supplementary material for: Bioinformatic Mining and Structure-Activity Profiling of Baeyer-Villiger Monooxygenases from Mycobacterium tuberculosis
Source: mSphere. 2022 Mar 17;7(2):e00482-21. doi: 10.1128/msphere.00482-21 (PMC9044951; doi:10.1128/msphere.00482-21)
Supplement: TABLE S2 [file msphere.00482-21-st002.docx]

**Table S2.**

|  | MymA | EthA | Rv0565c | Rv0892 |
| --- | --- | --- | --- | --- |
| CH | -5.5 | -5.7 | -5.1 | -5.9 |
| ThioA | -5.3 | -5.2 | -4.7 | -4.4 |
| 2-oct | -4.5 | -5.4 | -5.1 | -5.6 |
| Et3-ox | -5.0 | -6.2 | -5.4 | -5.6 |
