# Supplementary material for: Bioinformatic Mining and Structure-Activity Profiling of Baeyer-Villiger Monooxygenases from Mycobacterium tuberculosis
Source: mSphere. 2022 Mar 17;7(2):e00482-21. doi: 10.1128/msphere.00482-21 (PMC9044951; doi:10.1128/msphere.00482-21)
Supplement: TABLE S1 [file msphere.00482-21-st001.docx]

**Table S1.**

| Primers | Fwd | Rev |
| --- | --- | --- |
| ***Cloning in pLD1 vector*** *(NotI/NdeI, underlined)* | | |
| *rv0892* | 5'-ATATAGCGGCCGCAATGACCGGGCGATGTCCGAC-3' | 5'-GCCGGCATATGTCAAGCGCTTTGAGGCCGACTAG-3' |
| *rv3083 (mymA)* | 5'-ATATAGCGGCCGCAATGAACCAGCATTTCGACGTC-3' | 5'-TAATTCATATGTCAGGCCGCCGCGTGGTCTTG-3' |
| *rv3854c (ethA)* | 5'-ATATAGCGGCCGCAATGACCGAGCACCTCGACG-3' | 5'-AAATCATATGCTAAACCCCCACCGGGGCAGG-3' |
| ***Cloning in pVV2 vector*** *(NdeI/HindII, underlined)* | | |
| *rv0565c* | 5 ́-AAACATATGATGAGCGTGACTCCAAACGC-3 ́ | 5 ́-AAAAAGCTTTCATGCCGCGCCGAACACC-3 ́ |
| ***Cloning in pET28a vector*** *(BamHI/HindII, underlined)* | | |
| *rv3083 (mymA)* | 5’- ACGCGGATCCATGAACCAGCATTTCGACGTC-3’ | 5’- ATTCAAGCTTTCAGGCCGCCGCGTGGTCTTG-3’ |
| *rv3854c (ethA)* | 5’-TGCAGGATCCATGACCGAGCACCTCGACGTTGTCAT-3’ | 5’- ACTCAAGCTTCTAAACCCCCACCGGGGCAGG-3’ |
